# Transplant Trial Watch

**DOI:** 10.3389/ti.2023.11574

**Published:** 2023-06-09

**Authors:** Simon R. Knight

**Affiliations:** ^1^ Oxford Transplant Centre, Churchill Hospital, Oxford, United Kingdom; ^2^ Centre for Evidence in Transplantation, Nuffield Department of Surgical Sciences, University of Oxford, Oxford, United Kingdom

**Keywords:** kidney transplant, simultaneous pancreas kidney transplantation, randomised controlled trial, systematic review, metabolic acidosis

To keep the transplantation community informed about recently published level 1 evidence in organ transplantation ESOT and the Centre for Evidence in Transplantation have developed the Transplant Trial Watch. The Transplant Trial Watch is a monthly overview of 10 new randomised controlled trials (RCTs) and systematic reviews. This page of Transplant International offers commentaries on methodological issues and clinical implications on two articles of particular interest from the CET Transplant Trial Watch monthly selection. For all high quality evidence in solid organ transplantation, visit the Transplant Library: www.transplantlibrary.com.

**Randomised Controlled Trial**

Sodium Bicarbonate for Kidney Transplant Recipients With Metabolic Acidosis in Switzerland: A Multicentre, Randomised, Single-Blind, Placebo-Controlled, Phase 3 Trial.

*by Mohebbi, N., et al. Lancet 2023; 401 (10376):557–567.*

## Aims

The aim of this study was to examine the effects of sodium bicarbonate treatment on graft function in renal transplant patients with metabolic acidosis.

## Interventions

Participants were randomised to receive either oral sodium bicarbonate or matching placebo.

## Participants

242 kidney transplant recipients with metabolic acidosis.

## Outcomes

The primary outcome was the estimated glomerular filtration rate (GFR) slope over a treatment phase of 24 months. Secondary outcomes were serum bicarbonate and pH, albuminuria, and mean daytime systolic and diastolic blood pressure.

## Follow-Up

24 months.

## CET Conclusion

This multicentre study from Switzerland investigated the effect of using sodium bicarbonate to correct metabolic acidosis on the graft function of stable renal transplant recipients. Recipients with a serum bicarbonate level of <22 mmol/L were randomised to oral sodium bicarbonate or placebo for 2 years. Despite adequate correction of metabolic acidosis in the treatment group, there was no difference in eGFR decline between groups, leading the authors to conclude that sodium bicarbonate supplementation to preserve GFR in renal transplant recipients is not recommended.

The methodology of the study is excellent, with centralised variable block randomisation and placebo-control. A modified ITT analysis is used including all patients who were randomised and attended a baseline visit. It should be noted that the mean serum bicarbonate level in both groups at baseline was only just below the lower limit of normal (∼21 mmol/L), leaving the possibility that greater benefit may be seen in patients with a more profound acidosis. However, this was not supported by prespecified subgroup analysis (albeit with more limited statistical power).

## Jadad Score

5.

## Data Analysis

mITT.

## Allocation Concealment

Yes.

## Trial Registration

ClinicalTrials.gov—NCT03102996.

## Funding Source

Non-Industry.

**Systematic Review**

Heparin Thromboprophylaxis in Simultaneous Pancreas-Kidney Transplantation: A Systematic Review and Meta-Analysis of Observational Studies.

*by Ai Li, E., et al. Transplant International 2023; 36:10442.*

### Aims

This study aimed to assess the effect of heparin thromboprophylaxis in simultaneous pancreas-kidney (SPK) transplantation, pancreas after kidney (PAK) transplantation and pancreas transplant alone (PTA).

### Interventions

A literature search was performed on PubMed, EMBASE, BIOSIS, MEDLINE, Cochrane Library and Web of Science. Two reviewers independently selected studies for inclusion and extracted the data. Risk of bias was assessed using the Methodological Index for Non-Randomized Studies (MINORS).

### Participants

11 studies were included in the review.

### Outcomes

Outcomes of interest were pancreas thrombosis during early post-transplant period, incidence of postoperative bleeding, pancreas graft loss due to thrombosis, acute return to the operating room, and units of packed red blood cells (pRBC) used.

### Follow-Up

N/A.

### CET Conclusion

This systematic review and meta-analysis investigated the role of heparin thromboprophylaxis in simultaneous pancreas-kidney (SPK) transplantation, pancreas after kidney (PAK) transplantation and pancreas transplant alone (PTA). Study selection and data extraction were performed in duplicate. Only 11 studies, all of which were retrospective, were included. However, all the included studies were considered high quality (MINORS score > 60%). The authors found that heparin thromboprophylaxis reduced early pancreas thrombosis and pancreas loss by over two-folds for SPK, PAK and PTA, without resulting in an increase in the incidence of bleeding or acute return to the operating room. Heterogeneity was high for some of the outcomes but was not explored. No adjustments for confounders were made in the analyses.

### Registration

PROSPERO—CRD42021260585.

### Funding Source

None.

## Clinical Impact Summary

Graft thrombosis is a recognised and feared complication of pancreas transplantation, resulting from a thromboinflammatory response and relatively low flow through the graft [1]. It is more frequently seen in circulatory death (DCD) grafts and following pancreas transplant alone (PTA) compared to simultaneous pancreas kidney transplant (SPK) [1, 2]. Most centres employ some form of anticoagulation protocol in the peri-operative period to reduce the risk of thrombosis, although exact protocols vary considerably, and the evidence-base is limited. Use of anticoagulation is often monitored and adjusted using measures such as the activated partial thromboplastin clotting time (APTT) or thromboelastogram (TEG), with limited evidence that TEG monitoring may be beneficial [3, 4].

In their recent systematic review, Ai Li et al. attempt to summarise the literature regarding heparin thromboprophylaxis following pancreas transplantation [5]. They identified 11 studies investigating heparin use in SPK and PTA recipients, of which just four were comparative and none were prospective. They conclude that heparinization significantly decreases the risk of early pancreatic thrombosis and graft loss due to thrombosis, with no evidence of increased bleeding or reoperation risk.

Whilst the limited amount of observational data published in the literature does appear to support this conclusion overall, there are significant limitations to this study. There is no randomised controlled trial evidence available, and very limited comparative data meaning that the authors resort to comparing single-arm observational data to the control cohorts of other studies. Given the differences in protocols and surgical techniques between centres, the validity of this is uncertain. Even in the four comparative studies, there is significant heterogeneity in treatment protocols and monitoring strategies, meaning that the optimum regimen is unclear.

The authors employ fixed effects methods in some of their meta-analysis. Given the heterogeneous and observational nature of the data, the assumptions of a fixed effects analysis are probably not met. Indeed, re-analysis using a random effects model increases uncertainty and loses the significant treatment effects seen in fixed effects analysis.

It is unlikely that there is enough equipoise to undertake a large RCT of heparin versus no heparin following pancreas transplantation as most centres now use some form of anticoagulation. However, there is scope for future studies to investigate the optimal protocol and monitoring strategy for anticoagulation, including the use of TEG monitoring.

### Clinical Impact

2/5.
